# Challenges to the surveillance of non-communicable diseases – a review of selected approaches

**DOI:** 10.1186/s12889-015-2570-z

**Published:** 2015-12-16

**Authors:** Mareike Kroll, Revati K Phalkey, Frauke Kraas

**Affiliations:** Institute of Geography, University of Cologne, Albertus-Magnus-Platz, 50923 Cologne, Germany; Division of Epidemiology and Public Health, University of Nottingham, City Hospital, Hucknall Road, NG5 1 PB Nottingham, UK

**Keywords:** Disease surveillance, Facility-based surveillance, Sentinel surveillance, Non-communicable diseases, Literature review, Low- and middle-income countries, High-income countries

## Abstract

**Background:**

The rising global burden of non-communicable diseases (NCDs) necessitates the institutionalization of surveillance systems to track trends and evaluate interventions. However, NCD surveillance capacities vary across high- and low- and middle-income countries. The objective of the review was to analyse existing literature with respect to structures of health facility-based NCD surveillance systems and the lessons low- and middle-income countries can learn in setting up and running these systems.

**Methods:**

A literature review was conducted using Pub Med, Web of Knowledge and WHOLIS databases to identify citations published in English language between 1993 and 2013. In total, 20 manuscripts met inclusion criteria: 12 studies were analysed in respect to the surveillance approach, eight supporting documents in respect to general and regional challenges in NCD surveillance.

**Results:**

Eleven of the 12 studies identified were conducted in high-income countries. Five studies had a single disease focus, three a multiple NCD focus and three covered communicable as well as non-communicable diseases. Nine studies were passive assisted sentinel surveillance systems, of which six focused on the primary care level and three had additional active surveillance components, i.e., population-based surveys. The supporting documents reveal that NCD surveillance is rather limited in most low- and middle-income countries despite the increasing disease burden and its socioeconomic impact. Major barriers include institutional surveillance capacities and hence data availability.

**Conclusions:**

The review suggests that given the complex system requirements, multiple surveillance approaches are necessary to collect comprehensive information for effective NCD surveillance. Sentinel augmented facility-based surveillance, preferably supported by population-based surveys, can provide improved evidence and help budget scarce resources.

**Electronic supplementary material:**

The online version of this article (doi:10.1186/s12889-015-2570-z) contains supplementary material, which is available to authorized users.

## Background

Non-communicable diseases (NCDs) are chronic conditions with rather slow progression and rarely completely curable. The four most common NCDs - cardiovascular diseases, cancers, chronic respiratory diseases and diabetes - amongst other factors are mainly caused by preventable behavioural risk factors such as tobacco and alcohol consumption, unhealthy diet and insufficient physical exercise [1]. In 2012, 68 % of the global deaths were attributed to NCDs [2]. While the NCD mortality in the European Region is estimated to remain constant, the greatest increase will take place in the South-East Asian Region, Africa and the Eastern Mediterranean Region [1]. The increase of NCDs in low- and middle-income countries (LMICs) is accelerated by population ageing and is driven by rapid and unplanned urbanization and changing lifestyles. In addition, several LMICs are struggling with high prevalence of communicable diseases and an overburdened health care system, aggravating the impact of NCDs, for example through premature deaths [1]. About 48 % of NCDs in LMICs occur amongst people under the age of 70, compared to 28 % in high-income countries (HICs) [2]. The WHO estimates the cumulative economic losses attributed to cardiovascular disease, diabetes, cancer and chronic respiratory diseases to surpass US$ 7 trillion over the period 2011–2025 under a business as usual scenario in LMICs [3].

Given their devastating health and socioeconomic effects, NCDs have gained increasing attention over the past decade in the international community. The UN High-Level Meeting of the General Assembly on the Prevention and Control of Non-communicable Diseases passed a Political Declaration on NCD prevention and control in 2011, emphasizing the need for NCD surveillance [4]. The goal of disease surveillance is to address a defined public health problem and to develop evidence-based measures to protect and promote population health [5]. It is defined as “the ongoing systematic collection, analysis and interpretation of health data essential to the planning, implementation, and evaluation of public health practice, closely integrated with timely dissemination of these data to those who need to know” [6].

The WHO has assessed the current capacity for NCD surveillance as inadequate in several countries [1]. Evidence from HICs indicates that interventions for most NCDs can be effective and implemented at a rather low cost [1]. However, the long-term nature and complex disease aetiology of NCDs demand a comprehensive and long-term health-system mediated response. Essential to this goal is accurate and sequential data for planning and evaluation. Therefore, the WHO developed a global action plan for the prevention and control of NCDs, particularly cardiovascular diseases, cancers, chronic respiratory diseases and diabetes [7]. The action plan identifies six objectives (Additional file 1), one of which is monitoring the trends and determinants of NCDs and evaluating progress in prevention and control. In order to attain these targets, the WHO suggests the following policy options [2, 7]: strengthen vital registration systems and cancer registries, integrate surveillance into national health information systems, undertake periodic risk factor surveillance, and strengthen technical and institutional surveillance capacities.

Against this background, the objective of the current review was to analyse existing literature with respect to NCD surveillance systems in HICs and LMICs. In view of the different surveillance approaches and to increase comparability, a focus was laid on health facility-based approaches which can continuously provide routine data on confirmed cases and other essential information. The aim was to identify lessons learned in setting up and running such systems especially in LMICs with a rapid increase in the NCD burden.

## Methods

A literature review was conducted between March and June 2014 in three databases to identify manuscripts describing experiences with NCD surveillance systems globally. MEdical Subject Headings (MESH) terms were applied for searches in PubMed and WHOLIS. The same or similar terms and free text phrases were applied as search items to the Web of Knowledge in combinations separated by Boolean operators. Additionally, the webpage of the World Health Organization (WHO) as key organization was searched for reports on NCD surveillance. MESH terms or key words were selected from the following groups of generic terms: disease surveillance (“public health surveillance”, “sentinel surveillance”, “epidemiology”, “population surveillance”, “epidemiological monitoring”), non-communicable diseases (“chronic disease”) with a specific focus on high burden diseases (chronic respiratory diseases, cardiovascular diseases and diabetes), health information systems (“information systems”, “hospital information systems”, “health information management”, “health information systems”, “management information systems”, “geographic information systems”, “integrated advanced information management systems”, “ambulatory care information systems”, “information management”, “automatic data processing”, “electronic health records”), urban health (“urban health services”, “hospitals, urban”, “urban health”, “population dynamics”, “urbanization”, “cities”, “demography”, “urban population”), and spatial and socioeconomic disease patterns (“spatio-temporal analysis”, “socioeconomic factors”, “health status disparities”, “population characteristics”). Search algorithms included terms related to disease surveillance with at least one of the other above mentioned groups. Due to the vast difference in the surveillance approaches and the different study design of the papers, it was decided to restrict the review to health facility-based approaches in order to increase comparability of the studies. A systematic review approach [8] was therefore dropped.

**Fig. 1:**
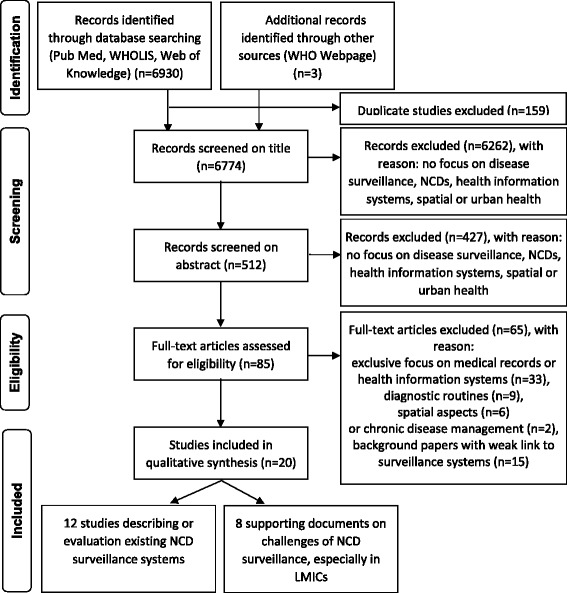
PRISMA flow diagram summarizing the literature search process

Inclusion criteria were set at full text citations published in English dated 1 January 1993 to 31 December 2013. After the identification of manuscripts, citations were archived in Endnote and selected by two independent reviewers in three steps (Fig. 1): title screening, abstract screening and full text review. Duplicates were removed electronically with a manual revision. Manuscripts were screened for the following inclusion criteria: those dealing with existing health facility-based disease surveillance systems AND focus on NCDs AND modes of data collection OR selection of reporting units OR approaches for data analysis OR role of private practitioners OR problems of data validity. Manuscripts only dealing with medical records or health information systems, diagnostic routines, or chronic disease management without link to disease surveillance and background reports with weak link to current surveillance systems were excluded. Bibliography of identified full text citations were screened for further relevant citations. Data on pre-identified variables were extracted in a pre-designed data matrix in Microsoft Excel™ 2011.

### Search results

The initial search identified 6933 potentially relevant published manuscripts, of which 159 duplicates were removed (Fig. 1). On the basis of the title, 512 manuscripts were selected for abstract screening, which 85 manuscripts were selected for full text review. Of these, 17 manuscripts met final inclusion exclusion criteria and were included in the review. In addition, three reports were selected from the WHO webpage.

Of the 20 citations, 12 manuscripts described or evaluated existing NCD surveillance systems (summary see Table 1), and eight supporting documents (summary see Additional file 1) provide background information on challenges to NCD surveillance in general and the status of NCD surveillance in LMICs. Since the 12 manuscripts predominantly describe approaches from HICs, the first part of the findings section provides an overview on NCD surveillance capacities in HICs and LMICs, mainly based on the eight supporting documents. The second part provides lessons learned on facility-based surveillance approaches mainly from HICs, based on the 12 identified manuscripts. The transferability of these approaches to LMICs and the combination with supporting approaches is addressed in the discussion section.

**Table 1:** Overview on manuscripts on NCD surveillance systems selected for the literature review

Authors & year	Country (region)	Surveillance approach	Data source/ reporting unit	Diseases under surveillance	Time	Lessons learned
Birtwhistle 2009 [17]	Canada	Longitudinal passive assisted sentinel surveillance system of NCDs (Pan-Canadian Primary Care Sentinel Surveillance Network, ongoing)	General practitioners (7 regional networks with ten practices in each network)	Hypertension, diabetes, depression, chronic obstructive lung disease, osteoarthritis	2008 (7 months) (first phase)	Primary care sentinel surveillance for NCDs is possible; major challenges are inclusion of risk factors and social variables, estimating practice denominators and ensuring representativeness of sentinel sites.
Bollag 2009 (BAG 2014) [18]	Switzerland	Longitudinal passive assisted sentinel surveillance system (Swiss Sentinel Surveillance Network, ongoing)	General practitioners, internists and paediatricians (total: 150–250 GPs)	Asthma, different communicable diseases	1989 – 2005	Sentinel surveillance on primary care level is a valid research instrument to analyse asthma incidence and seasonality. Denominator problems occurred since age and sex were only recorded for asthma cases, not all consultations.
Boydell et al. 1995 [19]	Northern Ireland	Cross-sectional pilot study on an active sentinel surveillance system (General Practice Data Retrieval Project)	General practitioners (*n* = 81) in 23 general practices (study population: 132,975)	33 chronic and acute conditions (results presented in paper: diabetes, myocardial infarction and depression)	1992–93	The accuracy of the diagnosis varied; validity of data needs to be explored in relation to the purpose for which it is to be used.
Deprez et al. 2002 [20]	USA (Maine)	Pilot study for a sentinel surveillance system using hospital data (passive), population-based phone survey and physician survey (active)	Secondary data: hospital admissions, emergency department/hospital outpatient data, physician survey (*n* = 59), population phone survey (*n* = 627)	Asthma	1994/95 (secondary data), 1997 (both surveys)	Data were useful to estimate the prevalence and to identify high risk groups; survey data provided otherwise unobtainable data on asthma symptoms; methods were not useful to identify environmental risks or the severity of asthma. The physician survey yielded useful information about diagnostic and treatment practices.
Fleming et al. 2003 [28]	Europe	Cross sectional survey, questionnaire based evaluation of sentinel surveillance systems (Health Monitoring in Sentinel Practice Networks Project)	Primary health care sector	33 sentinel practice networks, mainly on influenza, some also on diabetes	12/1998 – 12/2000	The primary care sector is an appropriate source for diabetes surveillance; if based on EMRs the costs of the system are very low; diagnostic validity of data has been demonstrated.
Klompas et al. 2012 [21]	USA (Massachusetts and Ohio)	Passive assisted sentinel surveillance system using the Electronic Medical Record Support for Public Health (ESP) surveillance platform	Primary health care sector (2 mixed provider groups: a multi provider multi-speciality ambulatory care provider group and a mixed inpatient and ambulatory provider group)	Diabetes, influenza, notifiable diseases	06/2006 – 07/2011	EMR based surveillance can provide timely and rich primary care data to public health departments on broad population and wide sets of health indicators; challenges include availability of sufficient electronic data, inclusion of contextual data, initial installation and activation of EMR based systems (financing) and electronic infrastructure to receive EMR-based reports.
Namusisi et al. 2011 [22]	Uganda (Mbarara district)	Pilot study on a passive assisted sentinel surveillance of NCDs	Regional referral hospital (*n* = 1) (1383 patient records)	Diabetes	01/2005 –04/ 2010	Use of hospital data is a valuable first step in setting up NCD surveillance systems, risk factor data are important for disease prevention and intervention. Incompleteness of records was a major limitation in the study.
Saran et al. 2010 [26]	USA	Pilot study for a passive national surveillance system	Various secondary data sets	Chronic kidney diseases (CKD)	10/2006 – 09/2008	Six broad themes, several measures for CKD and several data sources were identified for a pilot phase; active surveillance methods might be integrated in the future. Identification and acquisition of data sets and integration with other NCD surveillance systems were identified as some of the challenges.
Szeles et al. 2005 [23]	Hungary (4 counties)	Cross-sectional pilot study on a passive assisted sentinel surveillance system	General practitioners (*n* = 73) in four counties (Cohort size: 138,088)	Cardiovascular diseases, diabetes, liver cirrhosis, 4 malignant diseases	1998	Sentinel stations at primary care level are feasible and sustainable, data provide important information for health policy and health service planning, regular contact to reporting units is important.
Trepka et al. 2009 [24]	USA (Miami-Dade County)	Longitudinal pilot study for a passive assisted sentinel surveillance system (Miami Asthma Incidence Surveillance System)	Outpatient paediatric, allergy and pulmonary clinics (*n* = 18), emergency departments of hospitals (*n* = 3), standardized interviews with patients (*n* = 669)	Incident asthma	07/2002–06/2006	The pilot was useful in evaluating the case definition, in describing participants’ characteristics and health care use patterns; without mandatory laws, the system is not feasible.
Westert et al. 2005 [25]	Netherlands	Cross sectional surveillance study based on sentinel sites, health interviews with patients and census data (Dutch National Survey of General Practice 2)	General practitioners (*n* = 195) in 104 general practices (cohort size: 385,461), health interviews with Dutch speaking patients (*n* = 12,699) and non-native patients (*n* = 1339)	16 chronic conditions, e.g., BP, asthma/COPD, cancer, diabetes, myocardial infarction, vascular disorders, stroke, migraine	04/2000 –01/2002	Socioeconomic disparities existed in the 16 chronic conditions with higher prevalence rates in lower groups; the differences were similar using self-assessed health and practitioner data; this shows that accessibility to primary care is sufficient for all strata in the Netherlands.
Yiannakoulias et al. 2009 [27]	Canada (Alberta)	Study on passive surveillance using secondary data with special focus on spatial surveillance of NCDs using GIS	IPD hospital data, medical claims system (electronic public health insurance registry), hospital outpatient system	Asthma	1998–2005	The study reveals spatial differences in the asthma prevalence in Alberta. Disease distribution depends on case ascertainment algorithms and is aggravated through information inequity. Spatial data in surveillance are important to inform policy makers about disease patterns; however availability of spatial data is a limiting factor in many countries.

## Results

### NCD surveillance capacity in HICs and LMICs

Except one, all studies were conducted in HICs, i.e., in Northern America (Canada, USA) and Europe (Netherlands, Ireland, Switzerland, and Hungary). The limited number of publications on facility-based NCD surveillance systems in LMICs can be partly ascribed to the weak surveillance structures, as confirmed by the supporting documents: The WHO [1, 9] conducted questionnaire based surveys among its member countries in 2000, 2005, 2010 and 2013 in order to assess the national capacity for NCD prevention and control. It was found that some progress – mainly in HICs - has been made in the last decade. More countries have developed strategies for combating NCDs and created the necessary infrastructure. However, the implementation process in many countries was assessed as inadequate and strategies often exist mainly on paper. The survey in 2010 revealed that though more than 80 % of countries reported NCD mortality as part of their national health information systems, only 61 % of all countries said they had produced a report in the last three years; reporting in HICs was higher than in LMICs. HICs were 16 times more likely than LMICs to have population-based NCD mortality data in their national health information system. However, the quality and completeness of data was not assessed in the survey. Overall, a substantial proportion of countries, especially LMICs, have little usable mortality [10] and cancer registry data [11]. Routine facility-based data collection on NCDs is often not part of national health information systems [1]. According to the 2013 survey (complete results have not been published yet), only 42 countries had NCD surveillance and monitoring systems that enable reporting against the nine voluntary global NCD targets [2].

Alawan et al. [12] assessed the capacity of 23 LMICs to undertake surveillance using the same WHO data (2000 and 2010) and additional reports about data quality and judged the capacity of most of these countries as inadequate. Authors suggest, that major gaps exist in the accuracy, quality, standardisation of risk factor data, and reporting of NCD outcomes. Furthermore, data were often not linked to socioeconomic information and therefore did not facilitate the assessment of health disparities. Macfarlane [13] and Nolen et al. [14] also rated the quality of health data as inadequate in many LMICs. Macfarlane [13] identified costly duplications, inefficiencies and inconsistencies between institutions in the collection, reporting, storage and analysis of data as the main problems. Moreover, data were unreliable, unrepresentative and often not analysed and disseminated in a timely manner.

The member states of the WHO South-East Asia office for example stated in their regional meeting in 2012, that health system and surveillance capacities for addressing NCDs were poor due to negligence of NCDs over a long time [15]. A comprehensive framework, robust mortality data and sufficient funding to effectively plan and implement NCD prevention and control programs are missing in most countries in the region [16]. Therefore, the member states may need additional five years to establish robust surveillance systems and generate baseline data for targets of NCD monitoring due to the mentioned problems [15].

### Lessons learned with regard to facility-based NCD surveillance

The 12 studies provide important lessons learned in setting up and running health-facility-based NCD surveillance systems. The findings are summarized with respect to (1) surveillance approaches, (2) data sources, (3) data content, and (4) data analysis.

### Surveillance approaches

Nine studies can be categorized as sentinel surveillance systems [17–25]. Two studies represented passive surveillance systems based on secondary data [26, 27], and one [28] was a questionnaire-based cross-sectional survey on the activities of 33 sentinel surveillance networks in Europe (Table 1).

Five of the 12 studies reviewed maintained a single disease focus, three focused on multiple NCDs while the three others covered both communicable and non-communicable diseases (Table 1). Fleming et al. [28] identified different networks in Europe, e.g., on diabetes. The duration of the studies (Table 1) varied between seven months [17] up to five years [22]. Six studies were pilot studies [19, 20, 22–24, 26]. The Swiss Sentinel Surveillance System [18] and the Canadian Primary Care Sentinel Surveillance System [17] are the only ongoing routine surveillance systems.

### Data sources

#### Selection of data sources in passive surveillance systems

In a study from Canada, data were retrieved from an electronic public health insurance registry, which covers most permanent residents [27]. The registry data were linked through a unique identifier to other electronic health data sources, i.e., a medical claims and hospital systems. In another study from USA, data sources were selected using the Delphi method: first, all potential data sources were listed on the basis of literature and internet searches, second a standardized questionnaire was used to interview key informants to assess data accessibility and quality within the systems. Data from seven health care systems (e.g., private health care providers, laboratories, insurance data) and five non-health care systems (e.g., national surveys and registries) were initially included in the surveillance system [26]. Both studies exemplify the range of data sources that can be included and linked to form a comprehensive surveillance system, when available.

#### Selection of reporting units in sentinel surveillance systems

Seven out of nine sentinel systems used primary care providers as reporting units, i.e., general practitioners [17–19, 21, 23, 25, 28] (Table 1), one also included internists and paediatricians [18]. This indicates that NCD sentinel surveillance systems are increasingly applied at the primary health care level as general practitioners manage a large proportion of patients with NCDs [17, 23]. Furthermore, patients were not always referred to specialists unless complications arose [24]. The number of reporting units (Table 1) varied from one facility [22] to 104 [25].

Selection and stratification of the sentinel sites was based on different criteria: the geographic distribution [18, 23, 25], the settlement size, i.e., urban or rural setting [19, 20, 25], and the socioeconomic or demographic status of people in different areas [18–20]. Other criteria mentioned included the speciality of physicians [18], clinic type [25], availability of computer and commitment to participate [23], and the participation in a primary care network plus use of same electronic medical records (EMR) systems within this network [17]. Since selection criteria for reporting units are important to ensure representativeness of the data collected, context specific consideration of health-facility based, spatial and socioeconomic characteristics become vital.

#### Active components in sentinel surveillance systems

If there is no reporting obligation, approaching the reporting facilities actively may increase the reporting consistency. Five studies [17–19, 23, 24] mentioned that data reporting was voluntary. All nine sentinel studies were passive assisted or passive augmented systems with regular contact to the facilities. This included training [23], regular physical visits to the reporting units for problem solving and quality checks [19, 23], project meetings or workshops [19, 23] and regular feedback on reported cases and identified problems [18, 19, 23]. In Maine (USA), a postal physician survey was conducted on diagnostic and treatment patterns for asthma to verify disease detection algorithms [20]. In three of the nine sentinel studies [20, 24, 25] active surveillance components i.e., population-based surveys were linked to the facility-based surveillance approach.

### Data content

For diagnosis, three methods were identified for case verification: standardized diagnostic criteria [17–19, 22, 23], presumptive diagnosis based on clinical examination by a physician [17, 19] and disease detection algorithms [20, 21] (Table 2). Algorithms were also used to classify disease severity [20, 21]. If a patient was referred with a pre-diagnosed condition to the sentinel clinic, recording of the diagnosis without diagnostic criteria was also accepted [23].

**Table 2:** Data content: variables recorded in the studies

	Birtwhistle 2009 [17]	Bollag 2009 [18]	Boydell et al. 1995 [19]	Deprez et al. 2002 [20]	Fleming et al. 2003 [28]	Klompas et al. 2012 [21]	Namusisi et al. 2011 [22]	Saran et al. 2010 [26]	Szeles et al. 2005 [23]	Trepka et al. 2009 [24]	Westert et al. 2005 [25]	Yiannakoulias et al. 2009 [27]	No. of studies
Diagnosis	✓	✓	✓	✓	✓	✓	✓	✓	✓	✓	✓	✓	12
Age	✓	✓	✓	✓	✓	✓	✓	✓	✓	✓	✓	✓	12
Gender	✓	✓	✓	✓	✓	✓	✓	✓	✓	✓	✓	✓	12
Test results	✓					✓			✓	✓			4
Prescriptions	✓					✓				✓			3
No. of patient contacts		✓					✓	✓					3
Referral	✓					✓							2
Complications						✓							1
Address				✓		✓	✓		✓			✓	5
Risk factors	✓					✓	✓	✓					4
Obesity/BMI	✓					✓	✓						3
Smoking	✓						✓						2
Alcohol	✓						✓						2
Blood pressure						✓	✓						2
Family history							✓						1
Physical exercise	✓												1
Allergies						✓							1
Ethnicity						✓						✓	2

Since address is a personally identifiable variable and unavailable in majority of the records, the residence was recorded at the district [22], county [23], hospital service area [20] or municipality [21] level. The most common recorded risk factor was obesity (Table 2). The Electronic Medical Record Support for Public Health platform in Massachusetts and Ohio (USA) for example as the most comprehensive database extracted data on patient demographics, vital signs, diagnosis codes, test orders, test results, medical prescriptions, allergies, social history, and provider contact details [21].

Risk factors, comorbidities and complications are important parameters for NCD surveillance, but rarely collected as default data (Table 2). Furthermore, socioeconomic data which can help to identify health disparities, are rarely collected due to limited availability or lack of standardization in medical records. Nolen et al. [14] identified the following four general equity stratifiers: (1) socioeconomic position (measured through household wealth or assets; education or occupation are good indicators for socioeconomic position but no proxies for income or wealth), (2) gender, (3) ethnicity (religion, language spoken, migration background etc.), and (4) geographical area (urban vs. rural, better vs. worse-off areas). It is easier to collect these data if a standardized EMR system is implemented.

#### Population-based surveys as an additional tool

Population-based surveys were applied in three studies [20, 24, 25] to obtain more specific patient information. Surveys included questions on diagnosis, medication, symptoms, risk factors, health care utilization, disease knowledge, and socioeconomic and demographic characteristics of the respondents.

In two studies [24, 25], survey data were linked to the patients’ records of the sentinel sites through anonymous unique patient and practice identifiers. These studies show that the integration of population-based methods allows for the inclusion of much more detailed disease related information important for NCD management and socioeconomic characteristics of the patient. However, the approach requires informed consent of the patient, which can be time consuming and cost intensive.

The necessity to link different data sources to also capture socioeconomic and demographic data was also discussed by Macfarlane [13] who proposed to develop coordinated frameworks for collecting socioeconomic data from census, surveys, and routine databases and to ensure the collection and dissemination of disaggregated data at the local level. Health data and other administrative data can be linked through unique patient identifiers or – since unique identifiers are rarely available in LMICs - at least small-area identifiers (e.g., pin code).

### Data analysis

Data were usually automatically retrieved from electronic systems and transferred into a separate database [17, 20, 24]. In Uganda, data had to be manually entered from hospital records into an electronic database [22]. In the case of the Electronic Medical Record Support for Public Health platform (ESP), the software loaded EMR data from clinicians’ systems, analysed these data automatically using disease detection algorithms for events of public health interest, and electronically communicated findings to public health agencies [21]. Seven manuscripts described the calculation of prevalence and/or incidence rates for specific diseases according to gender and age distribution as the most important analytical outcome. The analyses focused on the exploration of seasonal patterns and time trends of asthma [18], spatial patterns of disease distribution [20, 21, 23, 27], and risk factor analysis for diabetes [22]. These case studies provide evidence that NCD interventions have to target specific socioeconomic, demographic and spatial population sub groups.

#### Challenges in analysing facility-based surveillance data

One major challenge for data analysis in facility-based surveillance is the availability of an appropriate denominator. In Hungary [23] and the Netherlands [25], citizens are registered to a specific general practitioner and the calculation of morbidity rates based on the catchment area is feasible. Five studies reported morbidity rates based on the total number of consultations per practitioner [18–20, 22, 24]. In Florida (USA) [24], the denominator (proportion of population served by each clinic) was ascertained from census data.

Five studies [20, 21, 23, 24, 27] mentioned case ascertainment bias, which can occur if the effectiveness for identifying cases is not the same for all population sub-groups or if the case definitions differ. Groups with lower socioeconomic status and migrants tend to have reduced access to formal health care systems, especially in countries without public health insurance. Prevalence and incidence rates for these groups are therefore often underrepresented. Comparison of subgroups is also difficult when the denominators are unavailable for these groups. Furthermore prevalence rates can be biased when case definitions are incorrectly applied by practitioners or when the case detection algorithms are inaccurate for the condition in question. Medical data sources, especially medical claims systems, can contain considerable diagnostic noise and miscoding [27]. Public health surveillance systems based on a single method of case ascertainment are likely to obscure differences between population sub groups for example with different socioeconomic background or different access opportunities to health care. Coding systems and the sources of diagnosis in data retrieval systems are therefore crucial for the internal data validity [23].

Yiannakoulias et al. [27] identified specific methodological challenges for using Geographic Information Systems (GIS). The small number problem (small stochastic differences in the number of cases resulting in large apparent differences in disease risks), multiple comparison problem (disease cluster detection methods are not designed to test explicit hypothesis about differences in absolute and relative risk from one region to another) and modifiable areal unit problems (challenge of choosing representative geographic regions or areas since results usually vary depending on the boundaries) which can bias the spatial distribution of cases.

Completeness of records as a challenge for data quality was addressed only by Namusisi et al. [22]. The authors recommended quality control checks (impossible values and internal consistency) during data collection and management, checking for very high or low reporting compared to the mean and the comparison with secondary data. Furthermore, it was observed that risk factor data were affected by social desirability bias, e.g., a patient does not admit smoking.

In summary, the problems illustrated by the studies demonstrate the difficulties in analysing data from non-standardized EMR systems and the importance of linking health data sets to other administrative data sources in order to generate essential and near accurate information.

## Discussion

Although lessons are rarely transferable directly from one setting to another, and especially from HICs to LMICs given the differences in health care system structures and capacities [29], the studies can be useful in guiding the design of new NCD surveillance systems. Since most LMICs are planning to establish routine NCD surveillance systems (e.g., WHO SEARO region [15]), the time is pertinent to incorporate information obtained from reviewing existing systems.

Facility-based systems offer many opportunities to collect a wide range of relevant information for NCD surveillance on a routine basis, but system requirements are high bearing the risk of distorted disease burden due to data quality issues through reporting inconsistencies. Though tertiary care hospitals, for example, often provide the infrastructure and personnel capacity for electronic health records, hospital data not collected for surveillance purposes may not be detailed enough to guide disease control decisions [30]. Passive facility-based NCD surveillance alone also requires necessary legal frameworks and capacity enhancement and are therefore less advisable. This especially applies to LMICs where the private sector plays an important role in the provision of health care but is largely unregulated [31, 32]. In India for example 69 % of the urban population prefer private over public health care facilities [33], but the involvement of private practitioners in current surveillance efforts is restricted to outbreak response. Therefore, regulation of the private sector and its integration in regular surveillance is an important challenge most LMICs face.

The review suggests that the sentinel surveillance approach is increasingly applied to NCDs since reporting of most NCDs is generally voluntary and complete case detection not required. This approach allows for the careful selection of appropriate reporting units to ensure data validity. The analysis of disease patterns and time trends of a representative population subgroup is sufficient as basis for health policy development and implementation. Findings also indicate that primary care level is adequate for the selection of reporting units since general practitioners manage a large part of patients particularly in LMICs. However, lack of EMRs, inadequate standardization and missing variables are major hindrances at this level. Setting up sentinel networks that use a single, standardized EMR system would be a solution since it increases the quantity, breadth and the timeliness of data. Furthermore, standardized data collection systems allow for the systematic collection of relevant information. The case sensitivity can be improved by applying disease detection algorithms in EMRs. However, diagnostic algorithms need to be updated regularly and there is no clear reference standard for the ascertainment of NCDs [30]. Alternatively, diagnostic guidelines can also help to ensure data quality.

Data collection from standardized medical records also allows for the systematic inclusion of other important variables for NCD control and evaluation of NCD care: co-morbidities, risk factors, critical events, hospital admissions and interventions. If standardized records are missing, this information can be obtained from encounter notes through templates. The studies also show that availability of a denominator is essential to calculate the prevalence of the population at risk and impedes data analysis. A sentinel system should therefore preferably include providers who can provide estimates for the denominators [24]. Finally, the integration of socioeconomic and spatial variables into collection routines is essential to facilitate equity-oriented decision-making and policy development [14, 15]. Geographic Information Systems (GIS), which are commonly used in communicable disease surveillance for outbreak control, can be a helpful tool for the documentation and analysis of the spatial distribution of disease patterns and risk factors [31]. However, the availability of spatial data is a limitation in LMICs [27]. The consideration of small administrative areas instead of accurate address might be a more feasible solution in these cases.

Systematic identification of reporting units is essential in a sentinel system to ensure representativeness of data. However, especially an EMR based system requires the appropriate electronic infrastructure to install EMR systems and health departments the adequate infrastructure to receive and analyse EMR based reports [21]. The availability of EMRs in clinics or the willingness to invest in and install an EMR system (computer or smartphone-based) could serve as an important criterion for the selection of reporting units in such a system.

Assisted or augmented passive sentinel surveillance helps to ensure regular reporting and to improve the data quality. Cooperation of the reporting units can be increased through a transparent reporting system, regular contact, workshops, trainings and feedback mechanisms. Assurance of data confidentiality, minimizing additional work and providing support during the start-up period are important aspects for increasing cooperation. Minor incentives such as a credit system providing benefits or alleviated access to support systems may help. The health department in Switzerland [34] for example offers free laboratory tests for selected diseases in laboratories owned by the health department as an incentive for its reporting units.

### Supporting approaches: population-based approaches

The review shows that linking facility-based surveillance systems with population-based approaches helps to obtain more specific information. Periodic surveys can provide otherwise unobtainable data on the prevalence of subclinical symptoms, risk factors, disease knowledge, socioeconomic data and health systems factors such as accessibility and health seeking behaviours. Furthermore, surveys help to overcome the problem of information inequity, i.e., information is often of lower quality in disadvantaged groups leading to distorted patterns of disease. This is especially the case in LMICs where not all groups will have uniform access to health care facilities due to financial constraints or spatial accessibility [35]. Therefore, some groups may be completely misrepresented in facility-based approaches. Since NCDs show slow disease progression, they are prone to be diagnosed at an advanced stage when the clinical symptoms become more prominent.

Large, regular, representative population-based surveys on NCDs have been conducted in HICs (e.g., phone based BRFSS in USA [36, 37]) and LMICs. Phone based [38] or house to house surveys (such as the Demographic and Health Survey (DHS) funded primarily by USAID [39]), institution based (e.g., the Global School-based student Health Survey (GSHS) [40] or the school-based behavioural risk factor surveillance system (SIVEA) [41]) have been conducted. The WHO STEPwise approach to chronic disease risk factor surveillance aims at collecting standardized data not only through questionnaires, but also includes physical and biochemical measurements. These allow for comparisons in time and across sites [42]. Data from 94 countries implementing the STEP surveys have been published and is available for review [43]. Surveys based on the STEPS approach (e.g., [44–46]) using physical and biochemical measurements help overcome social desirability (e.g., indication of weight) biases and underreporting of NCDs due to the lack of knowledge or nondisclosure [17]. A STEP based survey in Addis Ababa for example revealed a much higher rate of overweight (38 % versus 18 % versus) among women than the Ethiopian Demographic and Health Survey [47]. However, STEP surveys are costly and can only be carried out periodically. Alternatively, questions on NCD risk factors or symptoms could be added to on-going regular national census surveys [48].

Linking data from periodic population-based surveys and routine facility-based surveillance approaches can be challenging in the absence of unique identifiers. The identification of small-scale areas as a start seems to be a feasible solution [13, 14].

## Limitations of the study

The search was limited to English language only due to practical capacity constraints. The focus of the review was limited to facility-based surveillance and therefore other approaches such as vital registration systems or disease registries were excluded. Only selected NCDs with high disease burden were included in the review. We excluded neoplasms because of their near exclusive focus on cancer registries and clinical trials. The comparability of the studies was restricted as they did not always explicitly describe the data and exact variables which were collected. Due to these reasons, a systematic review approach was dropped.

## Conclusion

Surveillance of NCDs is essential, especially in LMICs in view of the increasing disease burden, the risk of comorbidities (e.g., diabetes and TB), and the long term socioeconomic impacts. So far, weak surveillance structures, lack of comprehensive and standardized electronic medical records, inadequate alternate data sources such as health insurance data and absence of unique identifiers to link different datasets hinder effective surveillance of NCDs in these countries. Adequate integration of the private sector in surveillance activities is also a major challenge.

Due to the complex system requirements, context specific multiple approaches have to be considered. The lessons learned from the review suggest that augmented sentinel surveillance at the primary care level can provide important information on the progression of NCDs on a routine basis. The introduction of a standardized EMR system could increase data availability and quality. Due to unequal access to health care especially in LMICs, periodic population-based surveys help to overcome health information inequity. They are useful in capturing additional variables otherwise difficult to capture in a facility-based system (e.g., health awareness, risk factors or prevalence of symptoms) and should be used. The information and knowledge gained through NCD surveillance is indispensable to manage NCD prevention and control across the globe and is a matter of urgency.

## Additional file

Additional file 1:
**Overview on supporting documents selected for the literature review [** 1 **,** 7 **,** 12 **–** 16 **,** 49 **].** (DOC 45 kb)
