# Supplementary material for: Challenges to the surveillance of non-communicable diseases – a review of selected approaches
Source: BMC Public Health. 2015 Dec 16;15:1243. doi: 10.1186/s12889-015-2570-z (PMC4682212; doi:10.1186/s12889-015-2570-z)
Supplement: Additional file 1: — Overview on supporting documents selected for the literature review [ 1 , 7 , 12 – 16 , 49 ]. (DOC 45 kb) [file 12889_2015_2570_MOESM1_ESM.doc]

**Additional file 1: Overview on supporting documents selected for the literature** review

| **Author & year** | **Objective** | **Major lessons learned for NCD surveillance** |
| --- | --- | --- |
| Alawan *et al.* 2010 [12] | The authors assess surveillance capacities in LMICs and outline a framework for NCD surveillance. | - LMICs face a rapid rise in NCDs, progress towards prevention has not kept pace due to wrong politics of priority setting. Monitoring is crucial for the information needed to develop NCD policies and programs and to assess these initiatives. A surveillance framework for NCDs, including a minimum of indicators covering exposures and outcome, is essential for policy development and assessment of disease trends. - The capacity to undertake surveillance was assessed in 23 LMICs (using WHO data from 2000 and 2010 and other published reports): The capacity of high-burden countries to effectively deal with the burden of NCDs was judged inadequate though the comparison of the surveys 2000 and 2010 shows some improvements. Major gaps existed in the accuracy, quality, standardisation of risk factor data, and the reporting of NCD outcomes. - The authors proposed a national framework which covers: key risk factors (behavioural, dietary, physiological and metabolic factors), outcomes (morbidity and mortality), health system response interventions, and health system capacity with a set of indicators for each category. |
| Choi *et al.* 2008 [50] | The authors identify seven important themes for enhancing capacity for NCD surveillance and prevention. | The SCIENCE approach consists of the following components:   - Strategy: to promote NCD surveillance, e.g. marketing to get funding, change perceptions of decision makers to put NCD on the political agenda, develop a global Strategy (e.g. WHO STEPS) which helps countries to develop their own national strategies, - Collaboration: involve multiple stakeholder from government, scientists, NGOs etc. with a harmonization, not standardization, of interests, - Information: accurate, timely and accessible surveillance information to develop policies and programs through good frameworks (e.g. Canadian Health Information Framework) and continuity in data collection (e.g. US Behavior Risk Factor Surveillance System) in order to ensure consistency; therefore data should be linkable to older records, - Education: raise awareness and build capacities, e.g. training health staff, - Novelty: new ideas have to be developed and used, e.g. new technologies, new forms of illustration for awareness building, new models of rapid, flexible and cost-effective surveillance, - Communication: effective way to distribute information, putting science into policy and policy into action, and - Evaluation: assess the design, implementation, utility and effectiveness of NCD surveillance. |
| Macfarlane 2005 [13] | The author develops a framework for LMICs in which health data can be linked to social and economic data. | - Due to the MDGs the importance of development indicators is increasingly acknowledged; however, the quality of health and development data is poor in many LMICs; costly duplications, inefficiencies and inconsistencies between institutions in the collection, reporting, storage and analysis of data pose problems; further, data are often unreliable, unrepresentative and note timely analysed. - It is required to develop coordinate frameworks for collecting and collating socioeconomic data from census, surveys, and routine databases and to ensure that the data can be disaggregated and disseminated for use at the local level; further, training for statisticians and information technicians is required since lack of human resources is a big challenge. |
| Nolen *et al.* 2005 [14] | The authors discuss opportunities to systematically identify and understand health inequities through health information systems in LMICs in order to support a culture of equity-oriented decision making and policy development. | - Health information systems in most LMICs don’t provide the necessary data for assessing health inequalities; at present, only 39 of 192 WHO member states, mostly industrialized countries, have a vital registration system coupled with a major household survey; in order to develop effective and long-term policies for the reduction of health inequalities, the continuous collection of health information data and equity stratifiers has to be implemented. - The following four general equity stratifiers should be considered: (1) socioeconomic position (measured through household wealth or assets, education or occupation are good indicators for socioeconomic position but no proxies for income or wealth), (2) gender, (3) ethnicity (religion, language spoken, migration background etc.), and (4) geographical area (urban vs. rural, better- vs. worse-off areas). - If data cannot be derived from the same source, it is needed to link health data with other administrative data (e.g. census, vital registration registers, household surveys). However, census data for example are usually only available on an aggregated level and don’t include unique identifiers. Therefore, a unique identifier is necessary, or at least a small-area identifier using administrative entities (e.g. pin code). |
| WHO 2011 [1] | The report describes the global burden of NCDs, of NCD risk factors, and the opportunities of tackling NCDs through NCD surveillance, population based prevention, strengthening health care and capacity building. | - Accurate national data are vital to reverse the global rise in death and disability from NCDs, therefore improving country-level surveillance and monitoring must be a top priority in the ﬁght against NCDs. - All countries should include three essential surveillance components: a) monitoring exposures (risk factors); b) monitoring outcomes (morbidity and disease-speciﬁc mortality, e.g. for cancers); and c) health system responses. - A survey among all WHO member countries in 2010 revealed that though more than 80% of countries reported NCD mortality as part of their national health information systems, only 61% of countries said they had produced a report on these data in the last three years; reporting in HICs is higher than in LMICs; further there is no information on quality and completeness of data. - HICs were 16 times more likely to have population-based NCD mortality data in their national health information system than LMICs. - Overall, a substantial proportion of countries, especially LMICs, have little usable mortality data and weak surveillance systems and data on NCDs are often not integrated into national health information systems. - However, signiﬁcant progress has been made over the past 10 years on risk factor surveillance, also in LMICs. |
| WHO 2013 [7] | The report introduces a road map and policy recommendations on how to control the global NCD burden (focus on cardiovascular diseases, cancers, chronic respiratory diseases and diabetes). | - The six objectives of the action plan comprehend priority setting on NCDs, national capacity building, reduction of exposure to NCD risk factors, strengthening national health systems, promoting research and monitoring the NCD burden and determinants and evaluate progress. - A framework is introduced including 9 voluntary global targets and 25 indicators to measure progress in NCD control. - The following actions are proposed for member states to fulfil the sixth objective (to monitor the trends and determinants of NCDs and evaluate progress): - Update legislation pertaining to collection of health statistics, strengthen vital registration and cause of death registration systems, to define a set of targets and indicators for NCD surveillance, - Develop and strengthen disease registries (e.g. on cancer), - Identify data sources and integrate surveillance into national health information system and undertake periodic risk factor surveys, - Strengthen technical and institutional surveillance capacities, - Report on a routine basis about trends in NCDs (morbidity, mortality by cause, risk factors and other determinants, disaggregated by age, gender, disability and socioeconomic groups), provide information about progress in NCD control, and - Increase and prioritize budget for NCD surveillance. |
| WHO 2012 [15] | The report evaluates the capacities of the South-East Asia countries to realize the implementation of the Political Declaration of the UN High-Level Meeting (UNHLM) on NCDs to prevent and control NCDs. | - NCDs have been neglected in the region for a long time. - Health systems in the South-East Asia region are generally weak when it comes to addressing NCDs, especially in the primary health care system. - Participants were concerned about the ability and capacity of existing surveillance mechanisms to generate the data needed for monitoring progress in achieving the proposed targets. - Currently, the baseline data for many targets are missing in many countries. The Region may need five years from now to establish a robust surveillance system to get a good baseline for the evaluation of progress in 2025. |
| WHO 2011 [16] | The report describes the current burden of NCDs, their risk factors and the national responses in South-East Asia. | - The WHO South-East Asia Regional Office states that lack of robust surveillance is an important barrier to effectively plan and implement NCD prevention and control programs in the region. Though most member countries have conducted NCD risk factor surveys, these are not yet routine and dependent on funds and other factors. - A comprehensive framework for NCD surveillance is missing, specific indicators and targets at the national level do not exist. Further, most countries don’t report reliable mortality data due to weak civil registration systems, and population-based cause-specific morbidity and mortality data collection systems continue to be poor. - There are no sufficient funds for NCD programmes in most countries despite the high disease burden. |
